# Editorial: Neurovascular dysfunction after stroke

**DOI:** 10.3389/fnmol.2022.1041551

**Published:** 2022-10-10

**Authors:** Qin Hu, Yi Yang, Zonguo Guo, Prativa Sherchan

**Affiliations:** ^1^Department of Neurosurgery, Ren Ji Hospital, Shanghai Jiao Tong University School of Medicine, Shanghai, China; ^2^Department of Neurology, the First Hospital of Jilin University, Changchun, China; ^3^Department of Neurosurgery, The 1st Affiliated Hospital of Chongqing Medical University, Chongqing, China; ^4^Department of Physiology, Loma Linda University, Loma Linda, CA, United States

**Keywords:** neurovascular dysfunction, stroke, hemorrhagic transformation, brain edema, blood–brain barrier

The pathophysiology of stroke is complex and is not limited to neuronal damage only. Neurovascular unit, refers to a group of closely related cells, including neurons, astrocytes, endothelial cells, pericytes and extracellular matrix components, is essential for the integrity of blood–brain barrier (BBB) and the maintenance of brain homeostasis. Neurovascular dysfunction, as a consequence of the impaired crosstalk between neurons, glia, and vascular compartments, is an early event following stroke (Schaeffer and Iadecola, 2021). Neurovascular dysfunction is critically involved in the development of hemorrhagic transformation, brain edema, neuroinflammation and cell death after stroke (Steliga et al., 2020). A better understanding of the molecular mechanisms underlying neurovascular dysfunction following stroke may have important implications for the treatment of brain injury after stroke. This Research Topic in Frontiers in Molecular Neuroscience focuses on recent advances in the pathophysiology, diagnosis, and potential therapeutic targets of neurovascular injury that occurs following stroke.

We first introduce 4 papers that stand their research on clinical observations. Zhang et al. firstly proposed that N-terminal pro-brain natriuretic peptide (NT-proBNP) levels could be used as a prognostic biomarker in patients with acute ischemic stroke treated with intravenous thrombolysis (IVT). They reported the association between NT-proBNP levels and hemorrhagic transformation in patients who have undergone intravenous thrombolysis, and an individualized prediction model was established based on NT-proBNP levels to predict the 3-month functional outcomes. In another retrospective study, Zhu et al. found that higher serum globulin levels were independently associated with hemorrhagic transformation (HT), and higher serum alkaline phosphatase and globulin levels were independently associated with a poor outcome in patients after IVT. Hyponatremia, one of the most common electrolyte disorders in stroke patients, is also found associated with HT and poor clinical outcome in patients with AIS who received thrombolytic therapy (He et al.). In subarachnoid hemorrhage patients, a comprehensive stratification and quantification of the immune infiltration status were performed by Wang X. et al. Key immune-related genes including SRPK1 and ZNF281 and immunomodulators CXCR1 and CXCR2 were observed to be involved in the progression of SAH (Wang X. et al.). These studies present exciting findings in the clinical stroke research and demonstrated potential biomarkers for evaluation neurovascular dysfunction and patient prognosis, especially in ischemic stroke patient treated with intravenous thrombolysis.

To investigate the mechanisms underlying neurovascular dysfunction following stroke, experimental models are still very important. The next 4 papers will introduce this aspect. Using transgenic galectin-7 mice, Wang M.-D. et al. determine that a significant relationship exists between human galectin-7 gene LGALS7 promoter region polymorphisms and the risk of intracerebral hemorrhage (ICH) and validated in Chinese Han population. And in β-catenin endothelial-specific conditional knockout (ECKO) adult mice, severe and widespread leakage of plasma IgG and albumin were observed in the cerebral cortex. The disruption of BBB in β-catenin ECKO mice may be caused by reduced expression of tight junction proteins levels in the brain endothelium, as well as increased endothelial vesicles and caveolae-mediated transcytosis through downregulating Mfsd2a and upregulating caveolin-1 expression (Hussain et al.). Xie et al. reported that Exendin-4 (Ex-4), a glucagon-like peptide 1 receptor (GLP-1R) agonist, preserve BBB integrity through GLP-1R/AMPK-dependent NF-κB/MMP-9 inhibition in subarachnoid hemorrhage rats, and could be a potential therapeutic target in SAH. In ICH mice, Ozanimod, a newly developed sphingosine 1-phosphate (S1P) 1/5 receptor agonist, was demonstrated to protect BBB and improve neurological function. The neuroprotective effect of Ozanimod might be related to reduce the density of activated microglia and infiltrated neutrophils in the perihematoma region.

Besides these original researches, several review manuscripts discuss a number of “hot topics” in neurovascular dysfunction, including pericytes (Zhou et al.), mitochondrial quality (Yang et al.), sex-associated differences (Tang et al.), eryptosis (Fang et al.), caspase-1 (Ye et al.), lipocalin-2 (Luo et al.) and semaphorins (Du et al.). Hopefully, studies addressing these topics will help in understanding the mechanisms underlying *Neurovascular dysfunction after stroke* and promote the translation in clinic.

## Author contributions

QH drafted the manuscript. YY, ZG, and PS revised it critically. All authors have approved the manuscript for publication.

## Conflict of interest

The authors declare that the research was conducted in the absence of any commercial or financial relationships that could be construed as a potential conflict of interest.

## Publisher's note

All claims expressed in this article are solely those of the authors and do not necessarily represent those of their affiliated organizations, or those of the publisher, the editors and the reviewers. Any product that may be evaluated in this article, or claim that may be made by its manufacturer, is not guaranteed or endorsed by the publisher.
